# A Rational Design of the Sintering-Resistant Au-CeO_2_ Nanoparticles Catalysts for CO Oxidation: The Influence of H_2_ Pretreatments

**DOI:** 10.3390/ma11101952

**Published:** 2018-10-12

**Authors:** Yuqi Sun, Wei Liu, Miao Tian, Liguo Wang, Zhongpeng Wang

**Affiliations:** School of Water Conservancy and Environment, University of Jinan, Jinan 250022, China; YuqiSun0821@163.com (Y.S.); tianm0821@163.com (M.T.); chm_wanglg@ujn.edu.cn (L.W.)

**Keywords:** H_2_ pretreatment, Au-CeO_2_ catalysts, CO oxidation, strong metal–support interaction, thermal-resistant

## Abstract

The redox pretreatment of samples is one of the crucial ways of altering the catalytic properties of the supported noble metal materials in many heterogeneous reactions. Here, H_2_-reducing pretreatment is reported to enhance the thermal stability of Au-CeO_2_ catalysts prepared by the deposition–precipitation method and calcination at 600 °C for CO oxidation. In order to understand the improved activity and thermal stability, a series of techniques were used to characterize the physico-chemical changes of the catalyst samples. H_2_ pretreatment may lead to: (i) a strong metal–support interaction (SMSI) between Au nanoparticles (NPs) and CeO_2_, evidenced by the particular coverage of Au NPs by CeO_2_, electronic interactions and CO adsorption changes. (ii) the production of surface bicarbonates which can accelerate CO oxidation. As a result, the H_2_ pretreatment makes the Au NPs more resistant to sintering at high temperature and enhances the CO oxidation activity. Furthermore, this reduction pretreatment strategy may provide a potential approach to enhance the thermal-stability of other supported noble metal catalysts.

## 1. Introduction

In heterogeneous catalytic reactions, the Au nanoparticles (NPs), as the active species with superior catalytic activities, have been attracted considerable interests in recent decades. They are active in a great deal of oxidation and reduction reactions, especially in the oxidation of CO [1]. Most intensely studied are not only Au/CeO_2_ catalysts, which have emerged as one of the best candidates for the low temperature CO oxidation [2,3], but also other catalysts such as Au/HAP [4], Au/Fe_2_O_3_ [5], Au/TiO_2_ [6] and Au/ZrO_2_ [7] have been investigated. However, the major problem of supported Au catalysts is the quick deactivation due to the low thermal stability during the catalytic reactions, thus, hindering their practical application. Au NPs are thermodynamically unstable and tend to be easily sintered when calcined at elevated temperatures or a long period of time. Since then, the active supported Au catalysts with the sintering-resistant property have been covered widely by numerous studies. As a result, it has been confirmed that the catalytic activity strongly depends on the change of Au particle size [8], the appropriate choice of support materials (oxides/non-oxides) [9], the formation of carbonates adsorbed on the active sites [10] and the metal-support interactions [11].

Several strategies have been developed to stabilize Au NPs for CO oxidation processes. Chen’s group reported that the enhancing of catalytic activity and the various carbonate species are contributed by the different small Au particle sizes [8]. Putla et al. introduced various dopants to Au/CeO_2_, and found that the appropriate support materials is able to improve the catalytic properties of Au based catalysts [12]. Qiao et al. demonstrated a classical strong metal–support interaction (SMSI) for Au/TiO_2_, which markedly depended on the reversible encapsulation of Au NPs by TiO_2_ support following high-temperature redox pretreatments [13]. Park’s group performed an oxidation atmosphere pretreatment on Au/TiO_2_ catalysts, and found that the oxidation pretreatment enhanced SMSI with the improved activity of CO oxidation [14]. However, the structural effects associated with the pretreatment process are not well understood. Because the Au NPs calcined at high temperatures are easily agglomerated after pretreatments [15], there are few efficient methods to improve the thermal stability of Au NPs with reduction conditions.

Here, inspired by previous research results, H_2_-reducing pretreatment method was used to fabricate SMSI in the Au-CeO_2_ catalysts further to trace whether the Au NPs can be stabilized while holding their high activity. So, in our study, the Au-CeO_2_ spheres calcined at 600 °C for various times, followed by pretreated upon H_2_ atmosphere at 200 °C. These products were characterized by X-ray diffraction (XRD), N_2_ adsorption–desorption, high-resolution transmission electron microscope (HRTEM), in-situ diffuse reflectance infrared Fourier transform spectroscopy (in-situ DRIFTS) and X-ray photoelectron spectra (XPS) to elucidate the effect of H_2_ pretreatments on the catalytic performance of CO oxidation. After H_2_ pretreatment, the introduction of SMSI in the Au-CeO_2_ catalysts could provide the enhanced activity and thermal stability until the calcination times increased to 12 h. Thus, the H_2_ pretreatment makes the Au NPs more resistant to sintering at high temperatures, which may be extended to other supported noble metal catalysts.

## 2. Experimental

### 2.1. Materials

Cerium nitrate (Ce (NO_3_)_3_·6H_2_O, 99.5%) was purchased from Shanghai Macklin Biochemical Reagent Factory, Shanghai, China. Polyvinyl pyrrolidone (PVP, K30), ethylene glycol and chloroauric acid (HAuCl_4_·4H_2_O, 99%) were obtained from Sino pharm Chemical Reagent Factory, Shanghai, China. All reagents were used without further purification. Deionized water and absolute alcohol were used throughout. 

### 2.2. The Synthesis of Au-CeO_2_ Samples

The CeO_2_ nanospheres were synthesized with minor alterations according to our previous reports [16]. In a representative experiment, Ce (NO_3_)_3_·6H_2_O (1.0 g) and PVP (0.4 g) were dissolved in ethylene glycol (30 mL) and distilled water (2 mL). The mixture was kept for another 20 min at atmospheric temperature. The resulting clear liquor was transferred to a 100 mL Teflon-lined autoclave and heated at 160 °C for 8 h. When the autoclave was cooled at atmospheric temperature, the mauve products were collected and washed separately with deionized water and ethanol in rinse-centrifuge cycles. The CeO_2_ products were dried at 60 °C in an oven overnight.

Au NPs were placed onto CeO_2_ by a deposition–precipitation method. At atmospheric temperature, 10 mL CeO_2_ precursor solution (0.145 mol/L) was added to 10 mL HAuCl_4_·4H_2_O (0.024 mol/L) whose pH was adjusted to 9 by NaOH (0.1 mol/L) in advance. The pH of the mixture solution was kept at ~9 for 1 h by NaOH (0.1 mol/L) at atmospheric temperature. Then the mixture was heated to 60 °C and stirred for 1 h. The precipitates were collected and washed with deionized water and dried at 60 °C for 12 h. The sample was calcined in a muffle furnace at 600 °C for 3 h, 6 h, 9 h, 12 h and 24 h. After that, the catalysts were pretreated at 200 °C for 0.5 h in H_2_ (5% H_2_ in N_2_) atmosphere, and denoted as AC-3-H, AC-6-H, AC-9-H, AC-12-H and AC-24-H, respectively. Apart from, the samples without pretreatment were denoted as AC-3, AC-6, AC-9, AC-12 and AC-24.

### 2.3. Characterization

Powder X-ray diffraction patterns (XRD) were carried on a BRUKER-AXS D8 Advance X-ray Diffractometer (Berlin, Germany) with Cu-Kα radiation (λ = 0.15418 nm) in the 2θ range from 10° to 90°. BET surface areas and size distribution of catalysts were determined by N_2_ adsorption-desorption isotherms using a Micromeritics ASAP 2020 surface area analyzer (Norcross, GA, USA). JEM-2100F microscope (JEOL Ltd., Tokyo, Japan) operating at 300 kV was employed to acquire high-resolution transmission electron microscope (HRTEM). The existence of surface elements and their valence states were confirmed by X-ray photoelectron spectra (XPS, Thermo Scientific Escalab 250Xi, Hongkong, China).

The in-situ diffuse reflectance infrared Fourier-transform spectra of CO adsorbed on solid samples were performed on a Nicolet IS50 (Thermo Fisher Scientific, Hongkong, China). Before the tests, the samples were pretreated at 200 °C for 0.5 h in H_2_ (5% H_2_ in N_2_) atmosphere. After cooling to atmospheric temperature, the background spectrum was recorded. Afterwards, a feed gas (0.2% CO/N_2_) was brought into the sample cell at the flow rate of 50 mL/min for 20 min and then the spectra were recorded until there was no variation inside. The spectra are displayed in Kubelka–Munk units, with the vertical axis implicating absorbance. 

### 2.4. Catalytic Tests

The activities of catalysts in CO oxidation were carried out in a fixed-bed quartz reactor with a length of 240 mm and a diameter of 8 mm. The catalyst weight was 50 mg, sieved into 40–80 mesh and the catalyst was not diluted in any diluent. A feed gas (0.2% CO in N_2_ + 5% O_2_ in He) was introduced into the catalyst equipment at the total flow rate of 100 mL/min, resulting in a space velocity (SV) of 120,000 mL·g_cat_^−1^·h^−1^. Then, the samples were heated from 30 °C at the rate of 4°/min. The products were detected with an online gas chromatograph (GC-2080, Ruipeng Chemical Instrument Corporation, Tengzhou, China) equipped with a thermal conductivity detector (TCD). The stability tests were measured with the same reactor and the same feed gas at 100 °C. 

## 3. Results and Discussion

**Figure 1:**
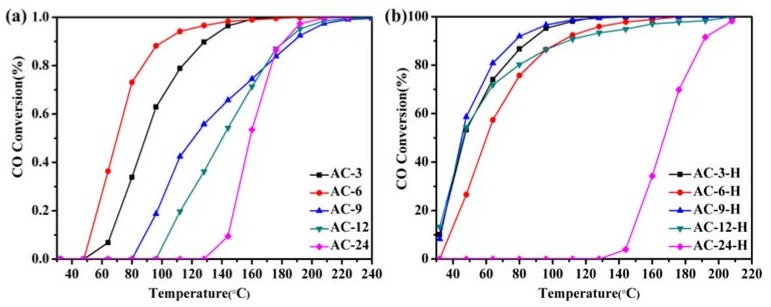
Catalytic performance in CO oxidation of Au-CeO_2_ with or without H_2_ pretreatments ((**a**): unpretreated; (**b**): H_2_).

Catalytic CO oxidation is used as a probe reaction to investigate the relations between the pretreatment conditions and the catalytic properties of the Au-CeO_2_ catalysts. Figure 1 describes the CO conversion profiles of the various Au-CeO_2_ samples calcined at 600 °C for 3–24 h. The T_50_ and T_90_ (temperatures for 50% and 90% CO conversion) of different samples were summarized in (Table S1) to compare the activity of the catalysts. For the Au-CeO_2_ samples calcined at different prolonged times without H_2_ pretreatments, the CO oxidation activities decrease in the order: AC-6 > AC-3 > AC-9 > AC-12 > AC-24 (Figure 1a). Obviously, the deactivation could occur in the Au-CeO_2_ samples without H_2_ pretreatment as the prolonged calcination time. Before the catalytic process, introducing the H_2_ pretreatments to the samples causes a significant enhancement of the catalytic activity (Figure 1b). The catalytic activity of AC-3 with uncovered Au NPs is significantly lower than that of AC-3-H with partially covered Au NPs. However, the AC-6-H sample (T_50_ = 60 °C, T_90_ = 106 °C) has a similar catalytic activity on AC-6 sample (T_50_ = 70 °C, T_90_ = 101 °C), indicating the active site of the AC-6 sample after H_2_ pretreatment is blocked. When the calcination time arises to 12 h, the samples with H_2_ pretreatment also provides stable catalytic performance (T_50_ = 46 °C, T_90_ = 111 °C). Especially, the AC-9-H sample has better catalytic activities, which gives the T_50_ of 45 °C and T_90_ of 77 °C.

**Figure 2:**
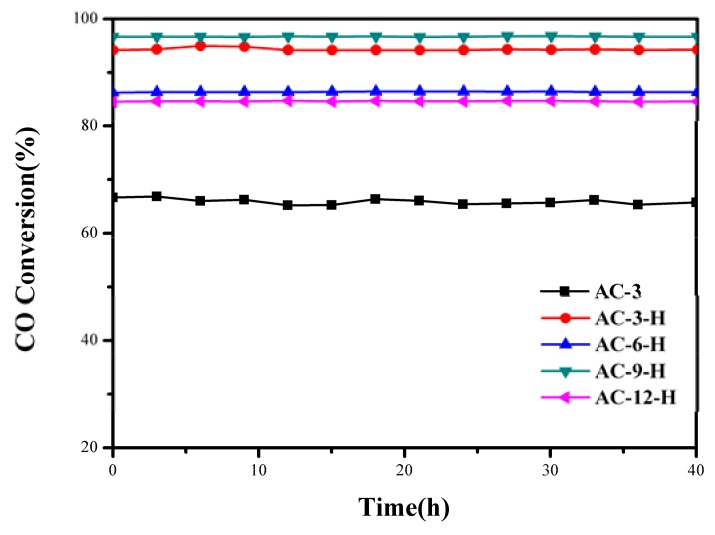
Stability tests of the Au-CeO_2_ with the different calcination times (3 h, 6 h, 9 h and 12 h) pretreated in H_2_ atmospheres at 100 °C for CO oxidation.

To better evaluate the thermal sintering performances, the temporal evolution profiles of the CO oxidation over the Au-CeO_2_ samples with and without H_2_ pretreatments (reaction at 100 °C for 48 h) are illustrated in Figure 2. There was no obvious deactivation in CO conversion, demonstrating its sintering-resistant catalytic performance. Overall, it can be found that H_2_ pretreatment could obtain thermally stable Au-CeO_2_ samples with a higher activity in comparison to samples without H_2_ pretreatments.

**Figure 3:**
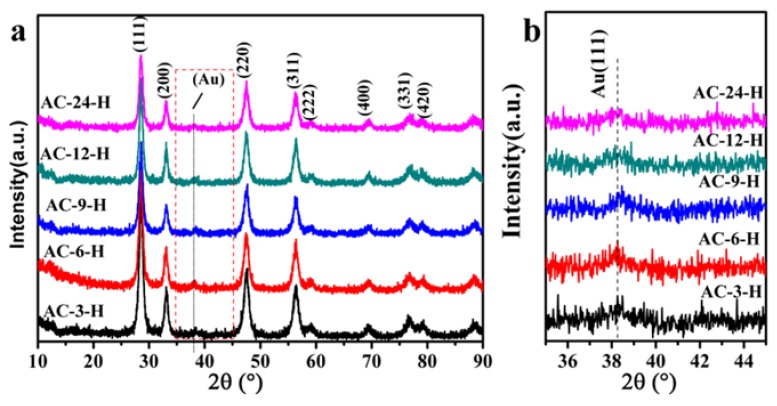
(**a**) X-ray diffraction (XRD) patterns of AC-3-H, AC-6-H, AC-9-H, AC-12-H and AC-24-H samples; (**b**) the enlargement of the box in (**a**).

The XRD profiles of different Au-CeO_2_ samples are shown in Figure 3 (with H_2_ pretreatment) and Figure S1 (without H_2_ pretreatment). The reference pure CeO_2_ sample in Figure S1 showed weak diffraction peaks with a lower average crystallite size of 5.0 nm (summarized in Table S2). All Au-CeO_2_ samples with or without H_2_ pretreatment clearly show obvious diffraction peaks at 2θ value of 28.5° (111), 33.1° (200), 47.4° (220), 56.3° (311), 59.0° (222), 69.4° (400), 79.1° (331) and 88.4° (420), which are the fluorite-type cubic structure of CeO_2_ [16]. Besides, the diffuse diffraction peaks of Au (111) located at 38.2°, suggesting the characteristic of Au NPs [17].

In order to know the influence of the calcination time on the textural properties of CeO_2_, average crystallite sizes were calculated from the Debye–Scherrer equation and specific surface areas were determined from N_2_ adsorption-desorption test (Table 1). It clearly shows that the specific surface area of Au-CeO_2_ samples is about 30–60 m^2^/g with pore volume of 0.14–0.20 cm^3^/g (Table S1). For the unpretreated Au-CeO_2_ samples, the prolonged calcination time is accompanied by the increase of CeO_2_ average crystallite size (Table S2). After H_2_ pretreatment, the CeO_2_ average crystallite size for all Au-CeO_2_ samples is decreased, implying that H_2_ pretreatment may restrain the size growth of CeO_2_. There is a decrease trend in surface area with the prolonged calcination time which correlates well with the increase of average crystallite size except the AC-9-H sample. The meticulous correlation of crystallite size and surface area of Au-CeO_2_ samples reveals the unusual crystallite size and surface area of AC-9-H sample. H_2_ pretreatment causes an obvious variation of average crystallite size for Au-CeO_2_ samples, which may lead to the unusual surface area for AC-9-H sample.

**Figure 4:**
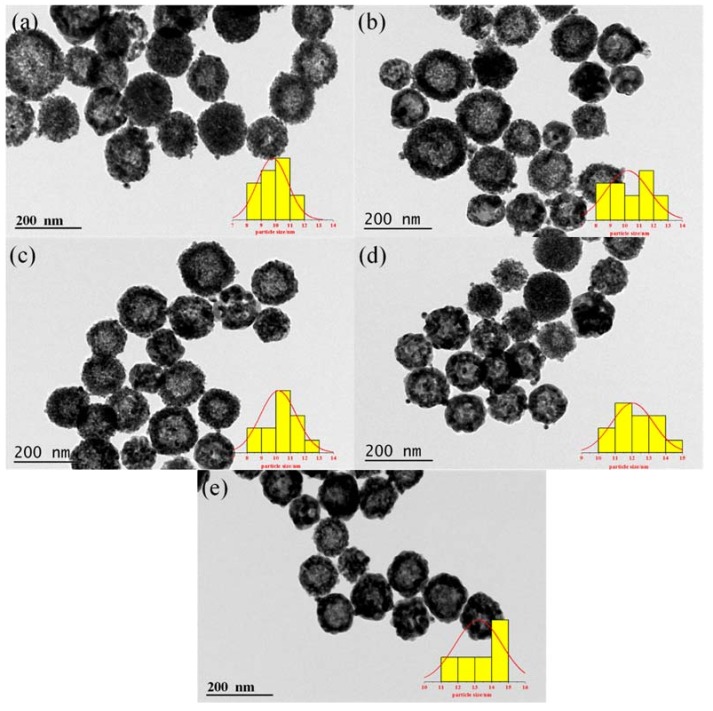
Transmission electron microscope (TEM) images of Au-CeO_2_ samples and the corresponding size distribution of the Au NPs. (**a**) AC-3-H, (**b**) AC-6-H, (**c**) AC-9-H, (**d**) AC-12-H and (**e**) AC-24-H.

Transmission electron microscope (TEM) is further used to identify microscopic structures of Au-CeO_2_ with or without H_2_ pretreatment. As shown in Figure 4, highly dispersed Au NPs are successfully attached on the CeO_2_ hollow nanospheres. For all Au-CeO_2_ samples, the CeO_2_ nanospheres have a particle size of ~120 nm (Table S3). With the increase of calcination time at 600 °C, there is no obvious agglomeration for CeO_2_ nanospheres. Significantly, it is clearly found that the sizes of Au NPs increase with the prolonged calcination time following in the order: AC-3-H (9.8 nm) < AC-6-H (10.1 nm) < AC-9-H (10.2 nm) < AC-12-H (12.0 nm) < AC-24-H (13.0 nm) (Table S3).

**Figure 5:**
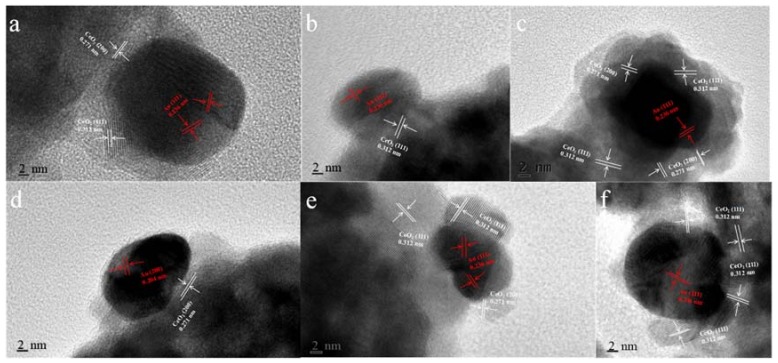
High-resolution transmission electron microscope (HRTEM) images of (**a**) AC-3, (**b**) AC-3-H, (**c**) AC-6-H, (**d**) AC-9-H, (**e**) AC-12-H and (**f**) AC-24-H.

As can be seen in Figure 5, the typical HRTEM micrographs of the single Au-CeO_2_ nanosphere reveal the presence of Au particles, in contact with the CeO_2_ surfaces. The fringe period of ~0.312 nm and ~0.271 nm is respectively in agreement with the (111) and (200) lattice spacing of CeO_2_, the lattice fringes of ~0.236 nm and ~0.204 nm are consistent with the (111) and (200) crystal plane of metallic Au. As shown in Figure 5a, Au NPs on the AC-3 sample without H_2_ pretreatment were naked. After H_2_ pretreatment, several important characters can be distinguished for the different samples in Figure 5b–f: (i) the naked and covered Au NPs coexist for AC-3-H, AC-9-H, AC-12-H and AC-24-H samples; (ii) whereas for AC-6-H sample, the coverage increased rapidly and the Au NPs are almost completely covered. Interestingly, AC-6-H is the only sample with Au NPs completely covered, which may be due to the increase of Au NPs and CeO_2_ crystallite size with the prolonged calcination time and/or the reduction of H_2_ pretreatment. The coverage of oxides support on metal NPs is a typical feature of SMSI phenomenon [13]. As a result, the coverage of CeO_2_ support on the Au NPs indicates the formation of SMSI in our Au-CeO_2_ catalysts with H_2_ pretreatments.

**Figure 6:**
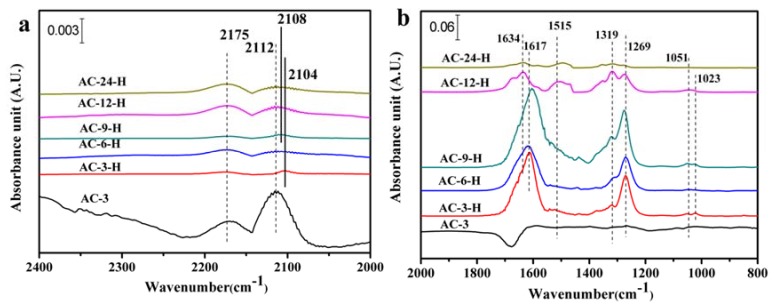
In-situ DRIFT spectra of CO adsorbed on Au-CeO_2_ samples at 25 °C after 20 min in the wavenumber region of 2400–2000 cm^−1^ bands (**a**) and 2000–800 cm^−1^ bands (**b**).

The SMSI generally results in fewer CO adsorption sites on Au NPs, because the CO adsorption sites are covered by the support layer [17]. The in-situ DRIFTS measurements of CO adsorption with a suitable probe molecule were used to explore the CO adsorption change and/or electron transfers of the Au-CeO_2_ catalysts. As shown in Figure 6a, two bands are detected on the 2168–2177 cm^−1^ and 2104–2112 cm^−1^ for all the samples. The former is assigned to gaseous CO [18], and the later can vest in CO at the metallic Au (CO-Au^0^) [19]. Obviously, the unpretreated AC-3 sample exhibits the highest peak intensities for the two bands with CO-Au^0^ centered at 2112 cm^−1^. Additionally, for the other unpretreated Au-CeO_2_ samples, the CO-Au^0^ band is also detected at around 2112 cm^−1^ (shown in Figure S2a). After H_2_ pretreatment, the CO-Au^0^ band for AC-3-H sample is blue shifted to 2104 cm^−1^ with the peak intensity largely decreased. As the calcination time increased, there is a blue shift for CO-Au^0^ absorbed species on Au-CeO_2_ samples, giving 2108 cm^−1^ over AC-9-H, and 2112 cm^−1^ over AC-6-H, AC-12-H and AC-24-H. After H_2_ pretreatment, the CO-Au^0^ band on Au-CeO_2_ samples is blue shifted, indicating more electropositive Au species can be formed on Au-CeO_2_ with H_2_ pretreatment. This blue-shift of the CO adsorption suggests the electron transfer from the CeO_2_ support to Au NPs [20]. Besides, the AC-6-H sample gives a lower intensity at the CO-Au^0^ band. According to the previous reports [17], the decreased CO adsorption is related to the loss in CO adsorption sites mainly deriving from the partial cover of Au NPs by CeO_2_ supports. In our work, the H_2_ pretreatment could be a powerful approach to alter the electron interactions between Au NPs and CeO_2_.

The in-situ DRIFT spectra of CO adsorption in the region of 2000–800 cm^−1^ (Figure 6b and Figure S2) shows bands corresponding to various carbonate surface substances, which are basically chemisorbed on CeO_2_. The presence of various carbonate species is summarized in Table S4. Further, Several important carbonate species can be distinguished for the Au-CeO_2_ catalysts: tridentate carbonates (bands at 1048–1073 cm^−1^ and 1266–1276 cm^−1^ and 1460–1550 cm^−1^), bidentate carbonates (bands at 1014–1028 cm^−1^ and ~1319 cm^−1^) and bicarbonate species (bands at 1600–1616 cm^−1^ and 1618–1638 cm^−1^) [21]. The negative bands at 1600–1700 cm^−1^ in Figure 6b and Figure S2b may be ascribed to the adsorption of water vapor on the sample before IR tests. However, only traces amount of tridentate carbonates and bidentate carbonates are formed on AC-3 sample. The carbonate surface species are more easily formed after H_2_ pretreatment. It can be noted from HRTEM results that the H_2_ pretreatment leads to a closer connection between Au NPs and CeO_2_ which facilitates CO oxidation at low temperatures [9]. Thus, the produced CO_2_ may be adsorbed on the ceria surfaces forming the large amounts of carbonates. When the Au-CeO_2_ samples are exposed to CO, the CO molecule can be adsorbed on the active Au NPs, forming CO-Au^0^ species. The lattice oxygen atoms on the surface of CeO_2_ are more reactive and can react with the adsorbed CO molecule, producing carbonates, biocarbonates species and oxygen vacancy. Finally, the surface carbonates species decomposed to CO_2_, with the remaining oxygen atom filling the vacancy. In fact, the proposed reaction mechanisms have already been widely accepted by other researches [22].

For the Au-CeO_2_ samples with H_2_ pretreatments, the band intensity of these carbonate species decreases in the order: AC-9-H > AC-3-H > AC-6-H > AC-12-H > AC-24-H. Among all samples with H_2_ pretreatments, AC-9-H and AC-3-H samples exhibit relatively larger band intensity of carbonate species, which may be ascribed to their higher surface areas compared with other samples. Significantly, bicarbonate species have been detected for all samples and account for the vast majority of these carbonates species. Moreover, when the calcination time increased to 24 h, the peak intensity of the bicarbonates species decreased sharply. It is well known [18,23] that the surface bicarbonates are less thermally stable than carbonates species, which facilitates faster desorption of CO_2_, allowing the surface effective for further chemisorption. Therefore, the presence of bicarbonates on the Au-CeO_2_ under H_2_ pretreatments can accelerate CO oxidation.

**Figure 7:**
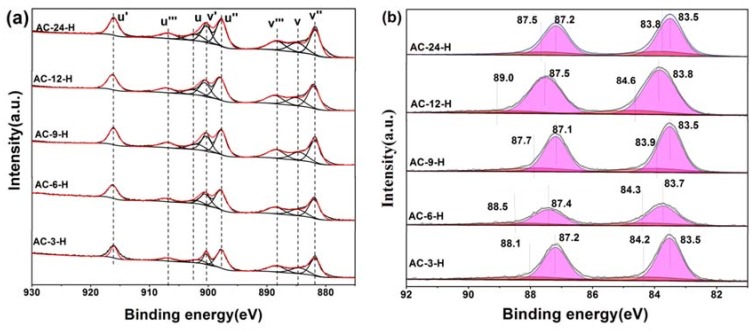
X-ray photoelectron spectra (XPS) of AC-3-H, AC-6-H, AC-9-H, AC-12-H and AC-24-H (**a**) Ce 3d; (**b**) Au 4f.

X-ray photoelectron spectroscopy (XPS) experiments were conducted in order to confirm whether there is a change in the valence states of the elements in the Au-CeO_2_ samples with or without H_2_ pretreatment. Figure 7 shows the XPS spectra of Au 4f and Ce 3d for different catalysts with H_2_ pretreatment, while Figure S3 shows the XPS spectra of AC-3 without H_2_ pretreatment. In the Ce 3d region, the peaks marked as u’ (916.0 eV), v’ (900.3 eV), u’’ (898.0 eV), v’’’ (882.0 eV), u’’’ (907.0 eV) and v’’’ (888.5 eV) correspond to the Ce^4+^ state, whereas that denoted as u (902.1 eV) and v (884.5 eV) are assigned to Ce^3+^ [24]. In Table S5, the results of the primary binding energies Ce 3d achieved by the XPS quantitative analysis, are reported for the different samples. There is no difference in the Ce 3d spectra of these samples except AC-6-H, indicating their similar electronic properties. Clearly, the over encapsulation of Au NPs for AC-6-H may result in a lower content of Ce^3+^.

In the Au 4f region, for quantitative evaluation, the Au 4f peaks with binding energies (BEs) were determined to be two different states. After curve fitting, the Au 4f peaks with binding energies of about 84.3 and 87.8 eV in AC-3 sample are attributed to the presence of Au^0^, while two weak BE peaks at 88.4 and 84.6 eV indicate the existence of the Au^δ+^ species [21]. After H_2_ pretreatment, a visible shift of BE to 83.5 eV–83.8 eV was observed on Ae-CeO_2_ samples, implying the Au NPs supported on CeO_2_ become electron-rich [11]. As a consequence of this electron-rich effect, the atomic ratio of the Au^δ+^ species for Au-CeO_2_ after H_2_ pretreatment decreases rapidly (Table S6). Overall, the electron transfers between Au NPs and CeO_2_ are created to make their interface more closely connected, leading to electron-rich Au after H_2_ pretreatment [4]. According to the in-situ DRIFTS, XPS and HRTEM results, the particular coverage of Au nanoparticles by CeO_2_, the electron transfers and the CO adsorption changes are identical to those characteristics in SMSI.

There are many efficient strategies for stabilizing Au NPs on various supports, such as the synthetic of yolk–shell nanoparticles [25], the coverage of Au NPs substrate coatings and the surface-modified of supports before Au NPs loading [26]. After H_2_ pretreatment, our CO oxidation results imply that the Au-CeO_2_ catalysts provide the enhanced activity and thermal stability until the elevated calcination times increased to 12 h. This is due to the occurrence of SMSI and the presence of bicarbonates. After H_2_ pretreatment, the construction of SMSI can be proposed for Au-CeO_2_ catalysts where the particular coverage of Au nanoparticles by CeO_2_, the electron transfers and the CO adsorption changes are in good agreement with those in classic SMSI. As for the non-linearity in the catalytic performance, the possible reason can be ascribed to the different coverage degree of Au NPs on the CeO_2_ support and the size variation of Au NPs. The partially exposed Au NPs for AC-3-H and AC-9-H, which are in direct contact with CeO_2_, the show the better catalytic activities for CO oxidation. On the contrary, the over-encapsulation of Au NPs on AC-6-H can suppress the active sites, resulting in relatively low activities. Moreover, the AC-24-H sample has poorer catalytic activities than others due to the relatively larger Au NPs. Besides, the surface bicarbonates would favor a faster desorption of CO_2_, through which the CO oxidation is accelerated. Moreover, the AC-24-H sample has poorer catalytic activities than others due to the relatively larger Au NPs and less bicarbonates surface species, which are still an important factor to limit the catalytic activity.

## 4. Conclusions

In summary, we have unambiguously demonstrated that the resulting Au-CeO_2_ samples calcined at 600 °C are highly stable under a H_2_ environment at elevated calcination times. Owing to the construction of SMSI and the presence of bicarbonates, it results in a remarkable high catalytic activity, which makes it possible to obtain the sintering-resistant Au catalysts. Furthermore, it is expected that these discoveries may provide a novel way for supported Au catalysts with thermal stability and may be expanded to other supported noble metal catalysts.

## Figures and Tables

**Table 1:** Structural information of the different samples.

Sample	CeO_2_ Average Crystallite Size (nm) ^a^	S_BET_ (m^2^/g) ^b^	Vp (cm^3^/g) ^c^
AC-3-H	10.2	55.1	0.20
AC-6-H	10.9	39.8	0.18
AC-9-H	10.0	45.0	0.14
AC-12-H	12.8	36.1	0.14
AC-24-H	13.3	33.2	0.18

^a^ Estimated from XRD studies; ^b^ Brunauer–Emmett–Teller (BET) specific surface area; ^c^ Total pore volume.

## Supplementary Materials

The following are available online at http://www.mdpi.com/1996-1944/11/10/1952/s1, Figure S1: (a) X-ray diffraction (XRD) patterns of CeO_2_, AC-3, AC-6, AC-9, AC-12 and AC-24 samples; (b) the enlargement of the box in (a), Figure S2: In-situ DRIFT spectra of steady-state CO adsorption after 20 min on Au-CeO_2_ samples at RT with the wavenumber region of (a) 2200–2050 cm^−1^ bands and (b) 2000–800 cm^−1^ bands, Figure S3: X-ray photoelectron spectra (XPS) of AC-3 (a) Ce 3d; (b) Au 4f, Table S1: T50 and T100 for Au-CeO_2_ with or without H2 pretreatment, Table S2: Structural information of the different samples, Table S3: Structural parameters of Au nanoparticles on various samples, Table S4: Assignment of different types of adsorbed carbonate species, Table S5: Relative content of Ce species for different catalysts obtained from Ce 3d XPS spectra, Table S6: Relative content of Au species for different catalysts obtained from Au 4f XPS spectra.

## References

[1] Abdel-Mageed A.M., Kučerová G., Bansmann J., Behm R.J. (2017). Active Au Species During the Low-Temperature Water Gas Shift Reaction on Au/CeO_2_: A Time-Resolved Operando XAS and DRIFTS Study. ACS Catal..

[2] Zhang X., Duan D., Li G., Feng W., Yang S., Sun Z. (2018). Monolithic Au/CeO_2_ nanorod framework catalyst prepared by dealloying for low-temperature CO oxidation. Nanotechnology.

[3] Ha H., An H., Yoo M., Lee J., Kim H.Y. (2017). Catalytic CO Oxidation by CO-Saturated Au Nanoparticles Supported on CeO_2_: Effect of CO Coverage. J. Phys. Chem. C.

[4] Zhan W., He Q., Liu X., Guo Y., Wang Y., Wang L., Guo Y., Borisevich A.Y., Zhang J., Lu G. (2016). A Sacrificial Coating Strategy Toward Enhancement of Metal-Support Interaction for Ultrastable Au Nanocatalysts. J. Am. Chem. Soc..

[5] Tanaka S., Lin J., Kaneti Y.V., Yusa S.I., Jikihara Y., Nakayama T., Zakaria M.B., Alshehri A.A., You J., Hossain M.S.A. (2018). Gold nanoparticles supported on mesoporous iron oxide for enhanced CO oxidation reaction. Nanoscale.

[6] Widmann D., Behm R.J. (2018). Dynamic surface composition in a Mars-van Krevelen type reaction: CO oxidation on Au/TiO_2_. J. Catal..

[7] Du C., Guo Y., Guo Y., Gong X.-Q., Lu G. (2017). Synthesis of a hollow structured core–shell Au@CeO_2_–ZrO_2_ nanocatalyst and its excellent catalytic performance. J. Mater. Chem. A.

[8] Chen S., Luo L., Jiang Z., Huang W. (2015). Size-Dependent Reaction Pathways of Low-Temperature CO Oxidation on Au/CeO_2_ Catalysts. ACS Catal..

[9] Tang H., Liu F., Wei J., Qiao B., Zhao K., Su Y., Jin C., Li L., Liu J., Wang J. (2016). Ultrastable Hydroxyapatite/Titanium-Dioxide-Supported Gold Nanocatalyst with Strong Metal–Support Interaction for Carbon Monoxide Oxidation. Chem. Int. Edit..

[10] Ke J., Xiao J.W., Zhu W., Liu H., Si R., Zhang Y.W., Yan C.H. (2013). Dopant-induced modification of active site structure and surface bonding mode for high-performance nanocatalysts: CO oxidation on capping-free (110)-oriented CeO_2_: Ln (Ln = La-Lu) nanowires. J. Am. Chem. Soc..

[11] Wang L., Zhang J., Zhu Y., Xu S., Wang C., Bian C., Meng X., Xiao F.S. (2017). Strong Metal–Support Interactions Achieved by Hydroxide-to-Oxide Support Transformation for Preparation of Sinter-Resistant Gold Nanoparticle Catalysts. ACS Catal..

[12] Sudarsanam P., Mallesham B., Reddy P.S., Großmann D., Grünert W., Reddy B.M. (2014). Nano-Au/CeO_2_ catalysts for CO oxidation: Influence of dopants (Fe, La and Zr) on the physicochemical properties and catalytic activity. Appl. Catal. B-Environ..

[13] Tang H., Su Y., Zhang B., Lee A.F., Isaacs M.A., Wilson K., Li L., Ren Y., Huang J., Haruta M. (2017). Classical strong metal–support interactions between gold nanoparticles and titanium dioxide. Sci Adv..

[14] Park E.D., Lee J.S. (1999). Effects of Pretreatment Conditions on CO Oxidation over Supported Au Catalysts. J. Catal..

[15] Cargnello M., Doan-Nguyen V.V., Gordon T.R., Diaz R.E., Stach E.A., Gorte R.J., Fornasiero P., Murray C.B. (2013). Control of metal nanocrystal size reveals metal-support interface role for ceria catalysts. Science.

[16] Liu W., Deng T., Feng L., Xie A., Zhang J., Wang S., Liu X., Yang Y., Guo J. (2015). Designed synthesis and formation mechanism of CeO_2_ hollow nanospheres and their facile functionalization with Au nanoparticles. CrystEngComm.

[17] Tang H., Wei J., Liu F., Qiao B., Pan X., Li L., Liu J., Wang J., Zhang T. (2016). Strong Metal-Support Interactions between Gold Nanoparticles and Nonoxides. J. Am. Chem. Soc..

[18] Wang W.W., Yu W.Z., Du P.P., Xu H., Jin Z., Si R., Ma C., Shi S., Jia C.J., Yan C.H. (2017). Crystal Plane Effect of Ceria on Supported Copper Oxide Cluster Catalyst for CO Oxidation: Importance of Metal–Support Interaction. ACS Catal..

[19] Wang S., Wang Y., Jiang J., Liu R., Li M., Wang Y., Su Y., Zhu B., Zhang S., Huang W. (2009). A DRIFTS study of low-temperature CO oxidation over Au/SnO_2_ catalyst prepared by co-precipitation method. Catal. Commun..

[20] Boronat M., Concepción P., Corma A. (2009). Unravelling the Nature of Gold Surface Sites by Combining IR Spectroscopy and DFT Calculations. Implications in Catalysis. J. Phys. Chem. C.

[21] El-Moemen A.A., Abdel-Mageed A.M., Bansmann J., Parlinska-Wojtan M., Behm R.J., Kučerová G. (2016). Deactivation of Au/CeO_2_ catalysts during CO oxidation: Influence of pretreatment and reaction conditions. J. Catal..

[22] Widmann D., Leppelt R., Behm R. (2007). Activation of a Au/CeO_2_ catalyst for the CO oxidation reaction by surface oxygen removal/oxygen vacancy formation. J. Catal..

[23] Davó-Quiñonero A., Navlani-García M., Lozano-Castelló D., Bueno-López A., Anderson J.A. (2016). Role of Hydroxyl Groups in the Preferential Oxidation of CO over Copper Oxide–Cerium Oxide Catalysts. ACS Catal..

[24] Venezia A.M., Pantaleo G., Longo A., Di C.G., Casaletto M.P., Liotta F.L., Deganello G. (2005). Relationship between structure and CO oxidation activity of ceria-supported gold catalysts. J. Phys. Chem. B.

[25] Lu J., Elam J.W., Stair P.C. (2013). Synthesis and stabilization of supported metal catalysts by atomic layer deposition. Acc. Chem. Res..

[26] Yan W., Mahurin S.M., Pan Z., Overbury S.H., Dai S. (2005). Ultrastable Au nanocatalyst supported on surface-modified TiO_2_ nanocrystals. J. Am. Chem. Soc..

