# Supplementary material for: A Rational Design of the Sintering-Resistant Au-CeO2 Nanoparticles Catalysts for CO Oxidation: The Influence of H2 Pretreatments
Source: Materials (Basel). 2018 Oct 12;11(10):1952. doi: 10.3390/ma11101952 (PMC6213528; doi:10.3390/ma11101952)
Supplement: Supplementary file 1 [file materials-11-01952-s001.pdf]

materials

Communication

A Rational Design of the Sintering-Resistant Au-CeO_2 Nanoparticles Catalysts for CO Oxidation: The Influence of H_2 Pretreatments

Yuqi Sun, Wei Liu *, Miao Tian, Liguo Wang and Zhongpeng Wang *

School of Water Conservancy and Environment, University of Jinan, Jinan 250022, China; YuqiSun0821@163.com (Y.S.); tianm0821@163.com (M.T.); chm_wanglg@ujn.edu.cn (L.W.)

* Correspondence: stu_liuw@ujn.edu.cn (W.L.); chm_wangzp@ujn.edu.cn (Z.W.); Tel.: +86-531-8276-9233 (Z.W.)

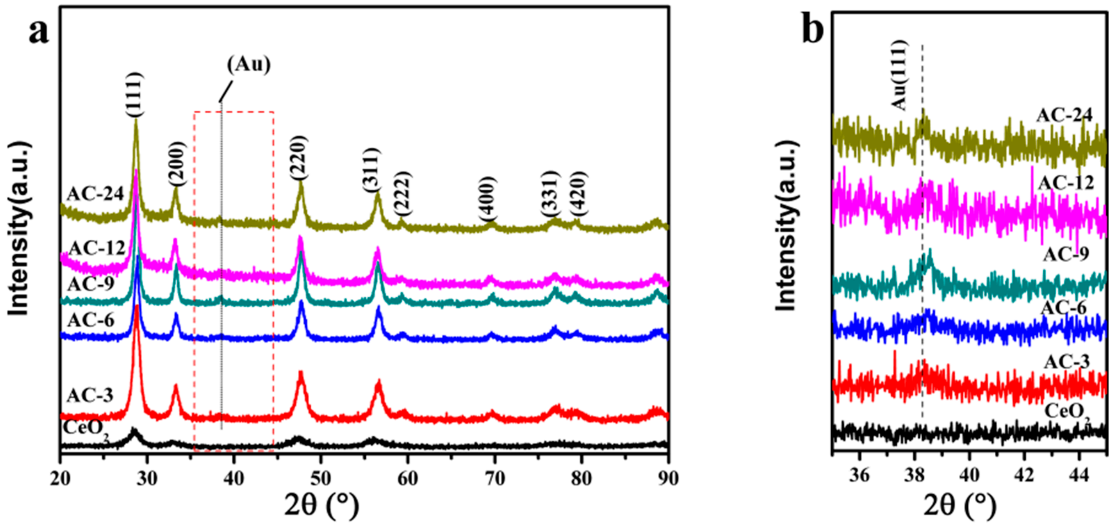

Figure S1. (**a**) X-ray diffraction (XRD) patterns of CeO_2, AC-3, AC-6, AC-9, AC-12 and AC-24 samples; (**b**) the enlargement of the box in (**a**).

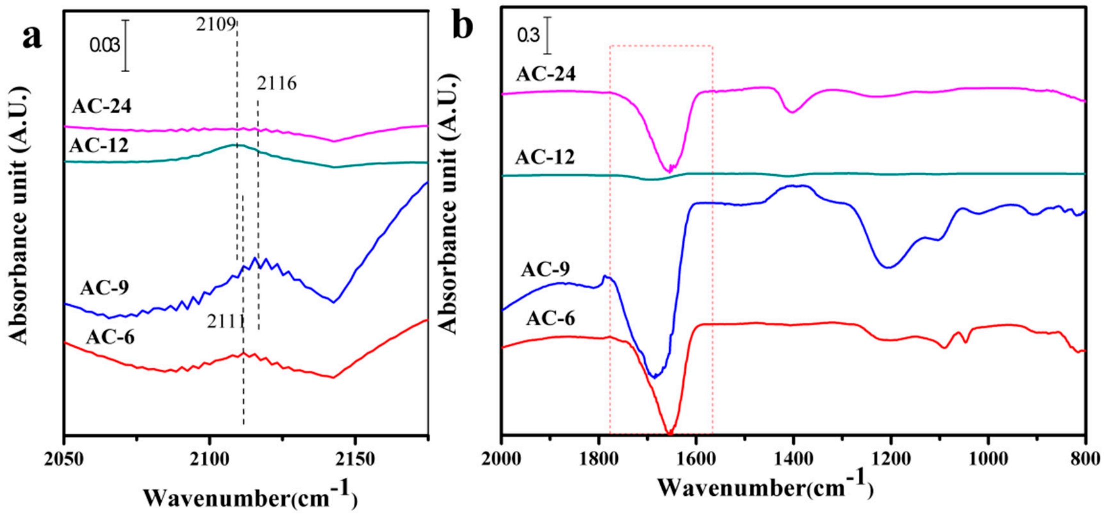

Figure S2. In-situ DRIFT spectra of steady-state CO adsorption after 20 min on Au-CeO_2 samples at RT with the wavenumber region of (**a**) 2200–2050 cm^{-1} bands and (**b**) 2000–800 cm^{-1} bands.

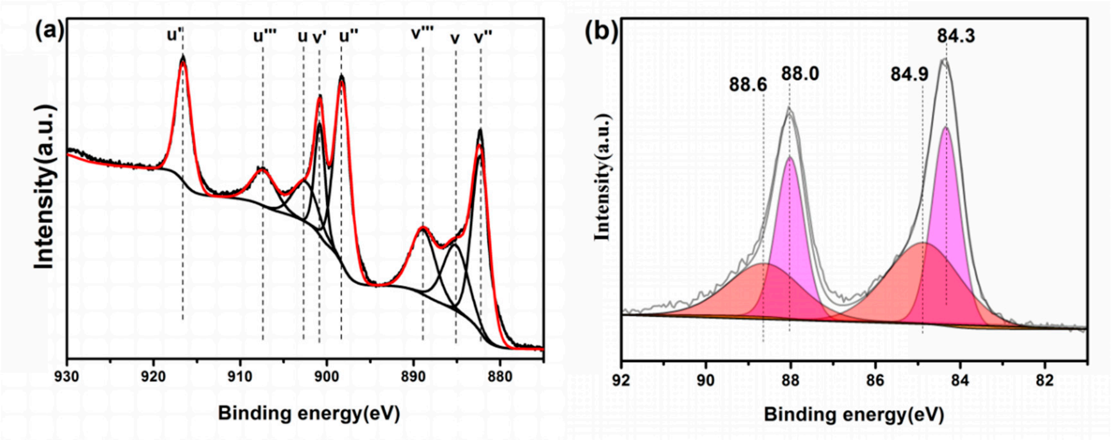

Figure S3. X-ray photoelectron spectra (XPS) of AC-3 (**a**) Ce 3d; (**b**) Au 4f.

Table S1. T_{50} and T_{100} for Au-CeO_2 with or without H_2 pretreatment.

Samples	Unpretreated		H_2 Pretreated	
	T_{50}	T_{90}	T_{50}	T_{90}
AC-3	89	128	46	86
AC-6	70	101	60	106
AC-9	120	188	45	77
AC-12	140	182	46	111
AC-24	159	182	167	192

Table S2. Structural information of the different samples.

sample	CeO_2 Average Crystallite Size (nm)
CeO_2	5.0
AC-3	11.1
AC-6	12.4
AC-9	12.7
AC-12	13.1
AC-24	14.6

Table S3. Structural parameters of Au nanoparticles on various samples.

Samples	Average CeO_2 nanospheres size (nm)	Average Au Nanoparticle Diameter (nm)
AC-3-H	121.8	9.8
AC-6-H	118.4	10.1
AC-9-H	120.2	10.2
AC-12-H	118.5	12
AC-24-H	120.1	13.2

Table S4. Assignment of different types of adsorbed carbonate species.

Species	Wavenumber(cm⁻¹)	Wavenumber(cm⁻¹) Pretreatment During CO Absorption					
		AC-3	AC-3-H	AC-6-H	AC-9-H	AC-12-H	AC-24-H
Tridentate carbonates	1048–1073<br>1266–1276<br>1460–1550	1051<br>1269	1051<br>1269	1051<br>1269	1051<br>1269	1051<br>1269<br>1515	1269<br>1500
Bidentate carbonates	1014–1028<br>~1319	1319	1319	1023<br>1319	1023<br>1319	1319	1319
Bicarbonate species	1600–1616<br>1618–1638	-	1617	1602	1613	1634	1634

Table S5. Relative content of Ce species for different catalysts obtained from Ce 3d XPS spectra.

Catalysts	Ce^{3+}		Ce^{4+}	
	Peaks	Content (%)	Peaks	Content (%)
AC-3	885.1<br>902.5	18.4%	882.4, 889.1, 898.3,<br>900.8, 916.6, 907.4	81.6%
AC-3-H	884.5<br>901.8	18.3%	881.8, 888.4, 897.7,<br>900.3, 916.0, 906.8	81.7%
AC-6-H	884.8<br>902.1	15.1%	882.0, 888.5, 897.9,<br>900.5, 916.2, 907.1	84.9%
AC-9-H	884.5<br>902.0	17.5%	881.8, 888.4, 897.8,<br>900.3, 916.1, 907.0	82.5%
AC-12-H	885.1<br>902.6	17.2%	882.1, 888.7, 898.0,<br>900.6, 916.3, 907.1	82.8%
AC-24-H	884.9<br>902.2	18.6%	881.8, 888.4, 897.8,<br>900.3, 916.0, 906.9	81.4%

Table S6. Relative content of Au species for different catalysts obtained from Au 4f XPS spectra.

Samples	Au^0			$Au^{\delta+}$		
	Peaks (eV)	content	FWHM (eV)	Peaks	Content	FWHM (eV)
AC-3	84.3<br>87.8	50.7%	0.78	84.6<br>88.4	49.3%	2.06
AC-3-H	83.5<br>87.2	76.8%	0.92	84.2<br>88.1	23.2%	3.36
AC-6-H	83.7<br>87.4	71.3%	1.11	84.3<br>88.5	28.7%	3.36
AC-9-H	83.5<br>87.2	79.1%	0.89	83.9<br>87.7	20.9%	2.72
AC-12-H	83.8<br>87.5	85.2%	1.19	84.6<br>89.0	14.8%	2.87
AC-24-H	83.5<br>87.2	78.9%	0.93	83.8<br>87.5	21.1%	2.3

© 2018 by the authors. Submitted for possible open access publication under the terms and conditions of the Creative Commons Attribution (CC BY) license (http://creativecommons.org/licenses/by/4.0/).
